# Nested and multipart prospective observational studies, flaming fiasco or efficiently economical?: The Brain, Bone, Heart case study

**DOI:** 10.1186/s12874-022-01675-w

**Published:** 2022-07-25

**Authors:** C. Christina Mehta, Kimberly S. Hagen, Lauren F. Collins, Renee’ H. Moore, Ighovwerha Ofotokun

**Affiliations:** 1 Division of Infectious Diseases, Department of Medicine, Emory University School of Medicine, 100 Woodruff Circle, Atlanta, GA 30322 USA; 2 Department of Behavioral, Social, and Health Education Sciences, Rollins School of Public Health, Emory University, 1518 Clifton Rd NE, Atlanta, GA 30322 USA; 3 Grady Infectious Diseases Program, Grady Health System, 341 Ponce De Leon Ave NE, Atlanta, GA 30308 USA; 4 Department of Epidemiology and Biostatistics, Dornsife School of Public Health, Drexel University, 3215 Market St, Philadelphia, PA 19104 USA

**Keywords:** Observational study, Study design, Nested study, Multipart study, Research methodology, Prospective study, MACS/WIHS Combined Cohort Study, Women’s Interagency HIV Study

## Abstract

**Background:**

Collecting new data from cross-sectional/survey and cohort observational study designs can be expensive and time-consuming. Nested (hierarchically cocooned within an existing parent study) and/or Multipart (≥ 2 integrally interlinked projects) study designs can expand the scope of a prospective observational research program beyond what might otherwise be possible with available funding and personnel. The Brain, Bone, Heart (BBH) study provides an exemplary case to describe the real-world advantages, challenges, considerations, and insights from these complex designs.

**Main:**

BBH is a Nested, Multipart study conducted by the Specialized Center for Research Excellence (SCORE) on Sex Differences at Emory University. BBH is designed to examine whether estrogen insufficiency-induced inflammation compounds HIV-induced inflammation, leading to end-organ damage and aging-related co-morbidities affecting the neuro-hypothalamic–pituitary–adrenal axis (brain), musculoskeletal (bone), and cardiovascular (heart) organ systems. Using BBH as a real-world case study, we describe the advantages and challenges of Nested and Multipart prospective cohort study design in practice. While excessive dependence on its parent study can pose challenges in a Nested study, there are significant advantages to the study design as well. These include the ability to leverage a parent study’s resources and personnel; more comprehensive data collection and data sharing options; a broadened community of researchers for collaboration; dedicated longitudinal research participants; and, access to historical data. Multipart, interlinked studies that share a common cohort of participants and pool of resources have the advantage of dedicated key personnel and the challenge of increased organizational complexity. Important considerations for each study design include the stability and administration of the parent study (Nested) and the cohesiveness of linkage elements and staff organizational capacity (Multipart).

**Conclusion:**

Using the experience of BBH as an example, Nested and/or Multipart study designs have both distinct advantages and potential vulnerabilities that warrant consideration and require strong biostatistics and data management leadership to optimize programmatic success and impact.

## Background

In 2018, the Specialized Center for Research Excellence (SCORE) on Sex Differences at Emory University proposed investigating whether estrogen insufficiency compounds the inflammatory and immune activation changes associated with chronic HIV-1 infection leading to end-organ damage and accelerated aging-related comorbidities affecting three organ systems: 1) neuro-hypothalamic–pituitary–adrenal axis (Brain), 2) skeletal (Bone), and 3) cardiovascular (Heart/vascular; BBH Study) [1]. Notably, the research within each organ system (brain, bone, heart/vascular) was envisioned as its own R01-level study (“Project”). At the time of study conception, Emory University had been a clinical research site for the Women’s Interagency HIV Study (WIHS) since 2013. Established in 1993, WIHS was the largest U.S.-based multisite prospective longitudinal cohort of sociodemographically-comparable women living with HIV or women at-risk for HIV. WIHS participants completed intensive semiannual study visits that included a comprehensive physical (including gynecologic) examination; a standardized interview of detailed health history, behaviors, and medication use; and laboratory testing for HIV viral load, CD4 count, complete blood count, and blood chemistry panels [2–4].

Given the availability and familiarity with the WIHS data and protocol and overlapping study populations of interest, each of the Brain, Bone, Heart/vascular projects was designed as a Nested sub-study of the Emory University WIHS clinical research site. In addition, since each of the Brain, Bone, Heart/vascular projects would be utilizing the same research participants and there would be partial overlap in data collected (notably estradiol, inflammatory biomarkers, basic demographic and clinical information) the projects were unified under a MultiPart study design. Thus, BBH Study design was finalized as a Nested, Tripartite prospective cohort study for the investigation of inflammatory comorbidities among women with HIV. Since an organizationally complex study design was chosen by BBH leadership and all Project Principal Investigators, the advantages and challenges were reviewed and discussed in order to adequately prepare for study implementation. Despite this review, there was little guidance available for key considerations of a Nested and/or Multipart study during the design phase or planning for implementation. To what extent, if any, do the design and implementation benefits of a) Nested studies and b) Multipart studies outweigh the design and implementation complexities of these study designs during the collection of new research data? As described below, we examined this methodological research question through a case study analysis of BBH. Our experience with BBH provides useful lessons for future researchers considering the use of either a Nested or Multipart study.

Observational study designs classically include cross-sectional, case–control, and cohort studies, although many related variations exist [5–7]. Optimal observational study design depends on many factors including budget and time constraints, the scientific question and study population, assessment and chronology of exposure and outcome [8, 9]. For studies collecting new data, the use of concurrent (survey, cross-sectional) or prospective (case–control, cohort) observational study designs can pose expensive and time-consuming obstacles to real-world implementation. Two study design approaches that may be leveraged to reduce the impact of these constraints include Nested and Multipart.

Nested studies hierarchically cocoon a proposed sub-study into an existing parent study. They are conducted concurrently and in conjunction with the parent study instead of after and/or separately. Multipart studies combine two or more operationally distinct but scientifically related observational studies into a single investigation composed of interrelated projects that utilize the same cohort of participants. Typically research study aims assess different aspects of an overarching research question. Multipart studies are similar but instead of one project with related aims, there are multiple projects with related aims both within and across projects. As a result, Multipart studies are substantially more organizationally complex than a single research study with multiple aims although there are economic and personnel benefits to consolidating as a Multipart study. Nested and Multipart study designs are not mutually exclusive and may be combined and utilizing them simultaneously can leverage the advantages of each, although this may come at a cost.

Despite growing use, information on the practical aspects of implementing Nested and Multipart prospective observational study designs for cross-sectional and cohort studies collecting new data are not well-described in the literature. A PubMed search of all fields conducted on 4/4/2022 comprising the terms “prospective” AND (“observational study” or “observational”) AND (“nested” or “hierarchical”) NOT (“case–control” or case control”) produced 524 results and another search of the terms “prospective” AND (“observational study” or “observational”) AND (“multi-part” OR “multipart” OR “multi part”) NOT (“case–control” or case control”) produced 125 results.

## Methods

BBH is a Nested, Multipart prospective cohort sub-study within the Emory University clinical research site of WIHS and thus includes women living with HIV and at-risk HIV-seronegative women. WIHS enrolled 4,982 participants from 10 sites over the course of 26 years with the goal of investigating the natural history of HIV in women [2–4]. Importantly, WIHS was broadly representative of the population of women living with HIV in the US and was a prospective cohort study with participants recruited directly from the community instead of a population registry or other established population sampling frame. Information on the BBH study design, proposed analytic plan, and power calculations are reported elsewhere [1]. Of note, BBH plans to recruit *n* = 120 women living with HIV and *n* = 60 at-risk HIV-seronegative women (total sample size *n* = 180) who agree to participate in all three interlinked BBH projects simultaneously. As a Nested prospective cohort sub-study within a prospective cohort study, all eligible Emory WIHS participants are approached for recruitment into BBH. Since participants in WIHS were recruited from the community and BBH participants are recruited from the Emory WIHS, no sampling strategy was implemented. Thus, the analytic plan for BBH did not require incorporation of sampling weights or other design elements, simplifying many aspects of design and statistical analysis. However, potential recruitment bias due to non-random participation into BBH is possible and may limit the generalizability of BBH to the parent WIHS population.

In 2019 with the support of the National Institutes of Health, WIHS merged with the Multicenter AIDS Cohort Study (MACS), a prospective longitudinal U.S. cohort of gay and bisexual men living with or at-risk for HIV established in 1984, to form the MACS/WIHS Combined Cohort Study (MWCCS), the largest and longest U.S. prospective observational cohort of men and women living with and without HIV. The overarching goal of MWCCS is to understand and reduce the impact of chronic health conditions occurring among people with HIV [10].

**Fig. 1:**
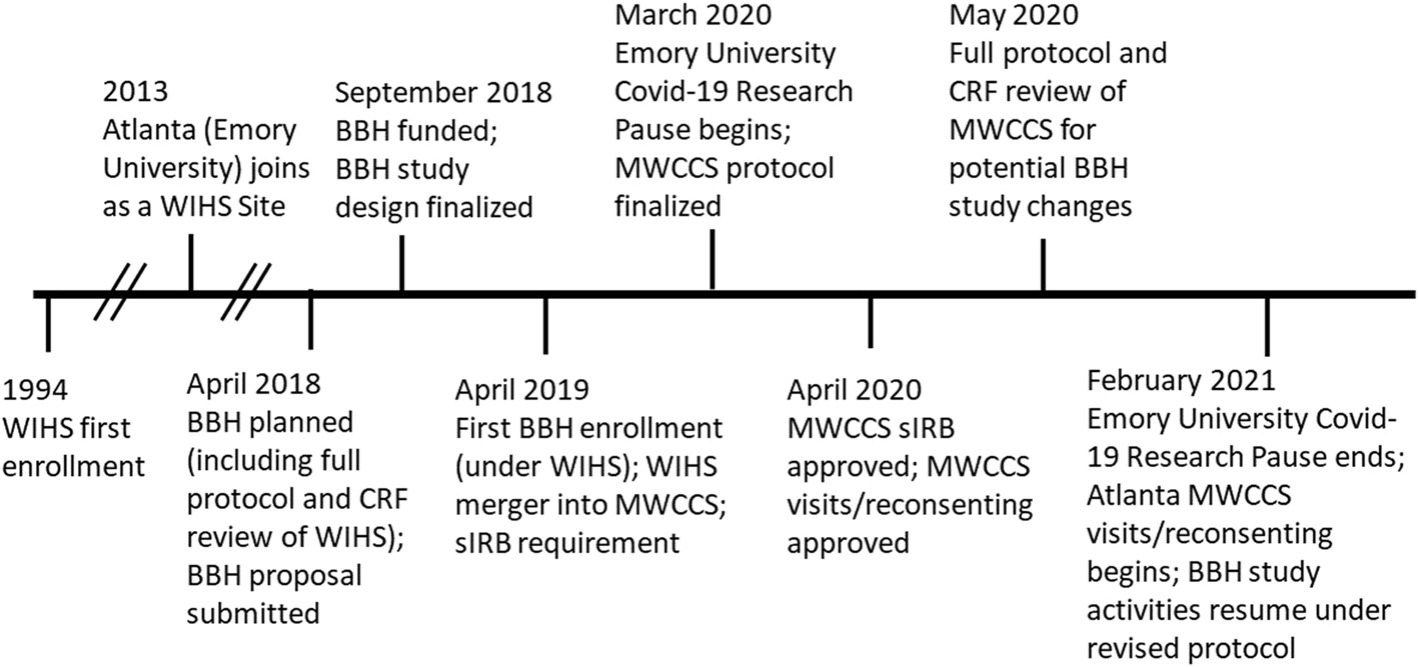
Brain, Bone, Heart (BBH) Study Timeline. Legend: BBH: Brain, Bone, Heart Study; MWCCS: Multicenter AIDS Cohort Study (MACS)/Women’s Interagency HIV Study (WIHS) Combined Cohort Study (MWCCS); CRF: case report form; sIRB: single Institutional Review Board

BBH was heavily affected when WIHS transitioned to MWCCS soon after the initiation of BBH participant recruitment in April 2019. Further, the impact of the COVID-19 pandemic on the implementation and productivity of BBH was significant and followed shortly after. In March 2020, Emory University instituted a mandatory COVID-19 research pause that halted BBH recruitment and in-person data collection activities for 11 months as shown in the BBH study timeline (Fig. 1). Due in part to these changes, recruitment for BBH was broadened to include eligible women outside of the original Emory University WIHS cohort in February 2022 making the study non-Nested for some BBH participants.

Since we use BBH as a case study on the benefits and complexities of Nested and Multipart study designs, where available, we provide summary statistics to compare different study design approaches (Nested, non-Nested).

## Results

Although BBH recruitment is on-going, we present information as of 3/9/2022. Of the *n* = 146 participants recruited into BBH, *n* = 131 (89.7%) were WIHS recruits, *n* = 6 (4.3%) are non-WIHS/non-MWCCS recruits, and *n* = 9 (6.2%) were new MWCCS recruits (not previously enrolled in the Emory University clinical research site of WIHS). From our experience with BBH enrollment and the study to date, we summarize our results and reflect on the advantages and challenges of Nested/Multipart studies through the lens of this Nested, Multipart prospective cohort study.

For non-WIHS/non-MWCCS participants recruited into BBH, the entry BBH interview lasts 1.5–2 h (extensive baseline questionnaires, blood work, and physical exam) compared to 25 min for WIHS and MWCCS participants (minimal baseline questionnaires). This is a considerable savings in participant and staff time as well as from reduced specimen collection. This experience highlights that a Nested study may require less resource investment than if it was conducted separately if a sub-study’s protocols can be adapted to the parent study’s infrastructure. Additionally, Nested studies that occur concurrently with the parent study can benefit in real time from reciprocated data sharing.

BBH investigators have frequently consulted with WIHS/MWCCS experts to gain their insight on a number of issues, most notably about previous estradiol and anti-Mullerian hormone (AMH) testing and interpretation as well as current estrogen research in the parent study. Nested study investigators may have ready access to additional expertise and collaboration with parent study investigators.

Using parent study data, BBH personnel were able to pre-screen WIHS participants for preliminary eligibility in BBH. Of *n* = 261 active Emory WIHS participants, *n* = 216 (82.8%) were preliminarily eligible, exceeding the BBH recruitment goal of *n* = 180. When recruiting outside of WIHS, research coordinators spent an additional 25 min in a telephone interview pre-screening potential participants, not including the additional time and effort dedicated to find and recruit these participants. In contrast, BBH enrollment by WIHS participants was high (61%, *n* = 131/216, with recruitment ongoing). Currently, no information is available on participant retention in BBH (study follow-up is on-going), although retention by parent study participation is of interest. Parent study participants recruited in to Nested studies may already be enthusiastic about additional research participation, be well retained over time, and have pre-collected data available to use in targeted participant recruitment and eligibility pre-screening.

The data manager, collaborative biostatisticians, laboratory scientists, overall BBH leadership are shared between the Emory WIHS, BBH, and across Projects for BBH. Shared study staff between the parent and sub-study has benefitted both studies by having personnel who are well-versed in both studies share knowledge and insight to smooth implementation, increased areas of synergy for efficient use of resources, and allowed for quick adaptations in protocol and data collection when required. Similar sharing of staff across Projects, reduced costs and gained time efficiencies through shared key study activities including: participant recruitment, scheduling, and tracking; specimen collection; laboratory testing; and, data processing, quality control, and analysis. We can roughly estimate that by combining tasks across projects, study staff effort has been reduced by ~ 20% compared to the needs from multiple, independent projects. As an example, assuming 10% full time equivalent (FTE) was needed for each individual Project for a shared position (30% FTE), across the entire BBH, 25% FTE was allocated. Notably, holding a monthly “all hands” BBH staff meeting has been extremely productive and directly beneficial to advancing BBH progress by allowing all team members (investigators, collaborative biostatical and data management teams, study coordinators, project leadership) to share information about the linked Projects, celebrate progress, and troubleshoot obstacles. This has increased the number of study staff who have a global view of the Tripartite study goals and trajectory and who are committed to overcoming any presented challenges.

Information required for more than one Project has been collected once and shared across Projects, which has reduced study participant time commitment and alleviated staff burden by eliminating redundancies in data collection, entry, cleaning, and reporting. Social demographics, basic medical history and medication use, inflammatory biomarkers, blood chemistry, viral load and other labs, are collected at the entry interview and shared across all three Projects. Without a shared entry interview, BBH participants and staff would need to conduct three entry interviews, one for each Project, potentially tripling the time and cost.

Despite thorough planning during the study design phase, numerous unexpected challenges arose during BBH implementation. First, given WIHS’ longevity (26 years), BBH’s study design was rooted in the assumption that its parent study would experience no more than occasional, temporary instabilities over the course of the BBH project period. That foundational conjecture was upended one year later when WIHS transitioned to MWCCS, requiring BBH to adapt to large changes in the parent study.

For example, BBH initially collected only BBH-specific dependent variables (neuropsychiatric testing, subclinical atherosclerosis evaluation, and osteoporosis/osteopenia assessment) and independent variable information (i.e., estradiol) while relying on the parent WIHS study for many sociodemographic, medical history, and clinical covariates. This was an efficient approach until WIHS was merged into MWCCS, as the MWCCS protocol differs substantively from the WIHS protocol (which was focused the impact and progression of HIV disease in women) in the following ways: scope (the MWCCS focuses on chronic conditions in older persons living with HIV), data collection (the MWCCS is tailored towards chronic illness and aging), and approach (the MWCCS comprises fewer study visits). Significant collaborative efforts among BBH investigators, biostatisticians, and study coordinators enabled a thorough review and prioritization of WIHS versus MWCCS data collection elements so that the robustness of the BBH data infrastructure could be protected. As a result, a comprehensive BBH protocol and case report form re-review was conducted to divide data elements extracted from the parent WIHS (now MWCCS) study into two categories: “Required,” and “Desirable if Available.” “Desirable if Available” elements included those deemed not crucial enough to the BBH study aims to warrant spending limited resources on capturing independently. “Required” elements were those that would need to be captured for all BBH participants, even if obtained independently from MWCCS at a significant time, personnel, and financial cost to the BBH. After review, *n* = 243 variables were designated as “Desired if Available” while *n* = 653 were designated as “Required.” Examples of “desired when available” included variables related to sexually transmitted infections, cancer history, and frequency of illicit drug use whereas “required” variables included current medications, lipid panel, physical examination, among others.

To take full advantage of cost-savings afforded by accessing data collected via its parent study, the original BBH protocol linked demographic and clinical data for each participant to their nearest WIHS semi-annual study visit. This protocol was founded on an expectation that all parent elements would have been collected for any BBH participant within a 6-month window of any given BBH study visit. Under the new MWCCS protocol however, tying a BBH study visit to the most recent MWCCS visit became administratively impractical because, in place of the WIHS model (single semi-annual study visit), MWCCS separates completion of survey/interviews, blood draws, and physical exam/study procedure components into individual visits that may be conducted in any order (per participant preference), at variable times, over the course of any given year. As a result, MWCCS data components available for any given BBH participant may have been collected anywhere from one week to one year before the next BBH visit. In response, the BBH protocol was amended to require each MWCCS individual study component be completed within one year prior to a BBH visit instead of 6 months prior to it.

In 2019 the parent MWCCS study became subject to the 2018 revised Common Rule requirement that a multi-institutional study use a single IRB (sIRB) to review research and be the IRB of record for all sites on the award. This led to numerous challenges associated with navigating the transition to a central coordinating center sIRB responsible for all 13 MWCCS sites across the country, with the untoward result that every sIRB requirement, complication, and delay affecting MWCCS also trickled down to affect BBH. Any time a Nested study is being incorporated into a larger infrastructure, there may be protocols for formal approval, project initiation, and periodic review that lengthen a Nested study’s timeline, although there still may be a time savings for Nested projects compared to designing and implementing a non-Nested study. However, notable exceptions can occur.

**Fig. 2:**
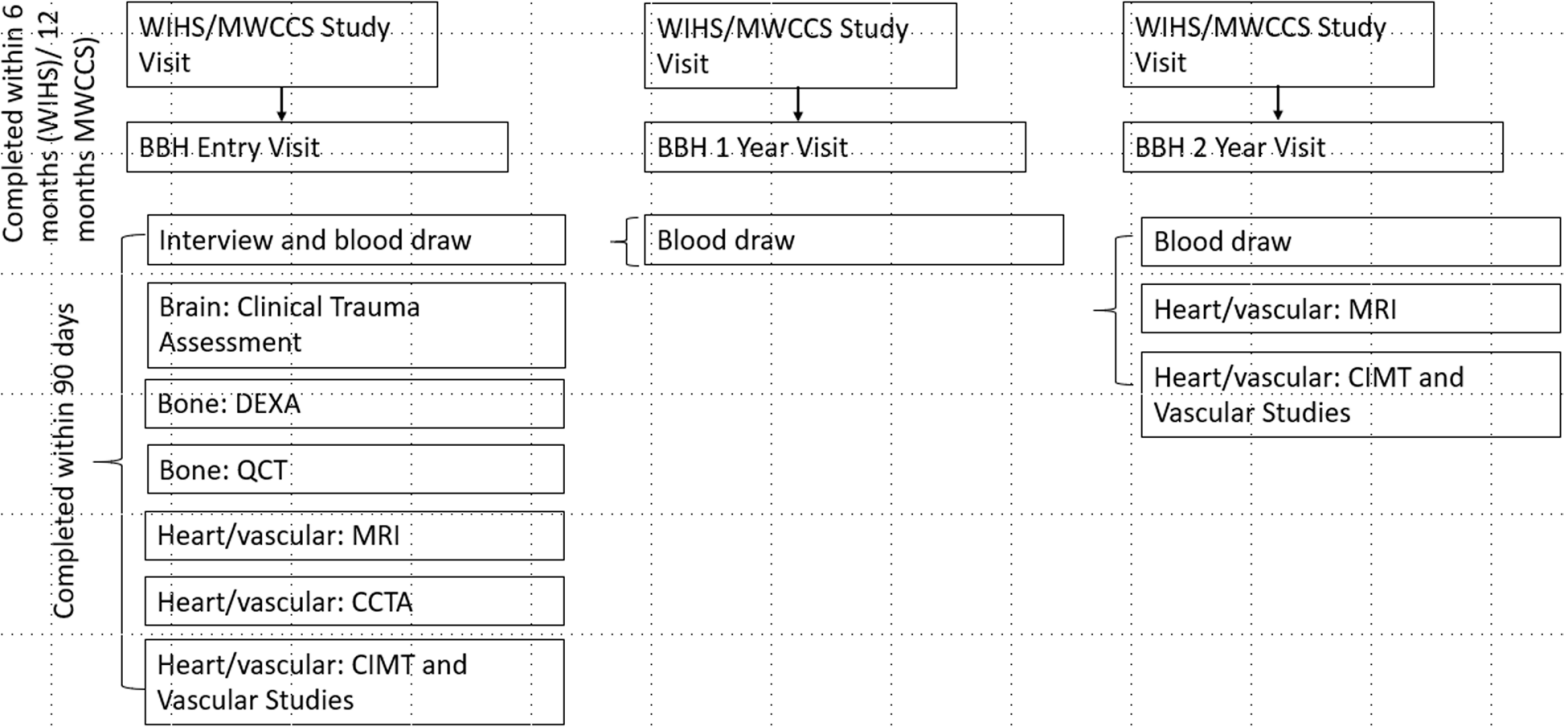
Brain, Bone, Heart (BBH) Study Visit Components and Visit Schedule [1]. BBH is a Nested, Multipart sub-study of WIHS, which later transitioned to MWCCS. BBH Study Visits are linked to the nearest parent study visit. Legend: BBH: Brain, Bone, Heart Study; MWCCS: Multicenter AIDS Cohort Study (MACS)/Women’s Interagency HIV Study (WIHS) Combined Cohort Study (MWCCS); DEXA: dual energy X-ray absorptiometry; QCT: quantitative computed tomography; MRI: magnetic resonance imaging; CCTA: coronary computed tomography angiography; CIMT: carotid intima-media thickness

In BBH, the inclusion and exclusion criteria are identical across all three linked Projects, so the pool of potential participants is restricted to that subset of individuals who meet every Project’s inclusion criteria and no Project’s exclusion criteria. This restriction provides the most economic efficiency by allowing the greatest overlap between data collection for Projects but does limit participation. In BBH, participant refusal to participate in the MRI portion of the Heart/vascular project has been a notable challenge. Lastly, BBH collects seven different required component interviews and procedures at the Entry Visit (Fig. 2), with the goal of thoroughly describing the simultaneous clinical characteristics of a participant’s baseline brain, bone, and heart/vascular organ systems. Due to the length of interviews and procedures, and the disparate physical locations of some of the procedural equipment, BBH participants required multiple appointments to complete all components of an Entry Visit. At the pandemic’s onset in March 2020, fewer than one-half of enrolled participants had completed all seven required Entry Visit components before the research pause began. After in-person research activities resumed 11 months later, the incompleteness of Entry Visit components for existing participants posed a potential challenge of BBH’s design to adequately answer the interlinked research questions. To compensate, the BBH protocol was revised to delink the three BBH Projects, except for when absolutely necessary to address a Project study aim. This allowed previously enrolled participants to re-engage in study activities so that missing components could be completed and critical interdependent components could be re-collected, as needed.

Importantly, none of the necessary changes to the BBH study protocol described above resulted in changes to the BBH project statistical analysis plans and sample size.

## Discussion

Leveraged funding and personnel optimization make Nested, Multipart, and Nested-Multipart studies an attractive option for conducting research that otherwise might not be economically or logistically feasible. Indeed, the economic advantage cannot be overstated, especially in light of budgets restraints and the difficulty in obtaining research grants large enough to support independent prospective observational cohort studies with new data collection. Although the advantages of these designs are readily apparent, the BBH experience illustrates that such designs may experience vulnerability if the study environment changes unexpectedly and/or in infrastructural ways. With concerted effort led by the biostatisticians, BBH has smoothly handled numerous unexpected challenges and, in the process, highlighted some considerations and recommendations useful for planning future Nested and/or Multipart studies, as described below.

### Nested study designs

There are two major factors to consider when planning a Nested study; 1) parent study stability and 2) parent study administration. Key questions to ask regarding stability include: “How long has the parent study existed?” and “Does the parent study have a history of adapting (or demonstrate the potential to adapt) its scope, data collection plan, participant eligibility, and/or study activities based on the evolution of its field?” While longstanding studies can experience episodes of instability, this is more likely in new studies that are becoming established, when even minor modifications can have significant ripple-effect consequences for a Nested study. The key questions to ask regarding administration encompass both policies and procedures. Specifically, “Are the regulatory needs of the parent and sub-study fully compatible?” and “Are the parent’s standard practices for data collection, management, analysis, and dissemination compatible with the anticipated operations of the sub-study?” A high level of administrative cooperation and organization between parent and Nested study leadership and staff is needed to ensure the smooth, scientifically rigorous, and administratively efficient operation of the Nested study. Ideally the Nested study research team will include a collaborative biostatistician who has a comprehensive understanding of both studies’ design so that the strongest aspects of the parent study can be incorporated into the sub-study’s design and processes for data collection, analysis, and interpretation of results.

### Multipart study designs

There are two major factors to consider when planning a Multipart study: 1) linked project study design, and 2) staff organizational capacity. Key questions to ask about linked project study design include: “To what extent can study activities be pooled or shared?” and “Where are the challenges in this study for study participants, study staff, and study budget, and can the order of study activities be organized to minimize these challenges?” The key question to ask about staff organizational capacity is: “Do we have sufficient staff time and expertise available to plan, administer, and maintain a complex data collection protocol?” The largest economic advantage of linked Multipart studies compared with multiple stand-alone investigations occurs when there is maximum overlap between projects. A study design that allows for pooling of related study activities yields the shortest time involvement, smallest resource use, and minimizes participant burden, thereby promoting retention and maximizing resources. Examples of pooled activities include collecting coordinated laboratory tests at a single visit and logically sequencing in-person study activities to minimize time and travel. These advantages are tempered by an organizationally complex study design, however, and require a well-trained central administration to ensure data collection across study components. Without a high level of attention to detail, the entire study integrity is subject to being compromised at the weakest link in the administrative chain. This becomes even more necessary when separate components of interrelated projects require field-specific domain expertise and specially trained staff. To optimize the success of Multipart studies, an exceptionally strong data and biostatistics team is recommended to ensure comprehensive participant tracking across all components, robust data management, routine quality assessments, and regular communication between and among project teams.

## Conclusion

Based on the BBH experience, investigators contemplating Nested designs should strongly consider the following recommendations, as they may have an important impact on the development of study protocol, sample size, and statistical analysis plans: 1) No matter how stable a parent study may appear, preparedness discussions should be prioritized at the design stage to identify – and, if possible, plan mitigation strategies for – project operations that are deemed to be particularly dependent on the operations of its parent study; 2) in advance, prioritize data elements extracted from the parent study as “Required” or “Desirable if Available” and plan for potential mitigation strategies should any element become unavailable; 3) be aware that any and all regulatory complications endured by a parent study have the potential to affect and delay a sub-study’s own protocol and operations; 4) be prepared that, in a fully Nested study, the sub-study protocol will need to navigate the delicate balance of simultaneous, and occasionally conflicting, pressures of economics, feasibility, and optimal scientific approach and 5) although random selection of substudy participants is recommended, if deemed impractical, thoughtfully consider the statistical and causal implications of potentially non-random substudy participant recruitment and enrollment from the parent study (ex [11, 12]).

Similarly, investigators contemplating Multipart study designs should strongly consider the following recommendations: 1) regular and direct communication between project-specific and shared study personnel (especially biostatisticians and investigators) can foster a more rapid response to challenges and facilitate innovation and adaption; 2) Multipart study designs require more intensive statistical collaboration than traditional designs; and, 3) in exigent circumstances, focus on preserving the critical linkages and delinking other data collection aspects as necessary.

In sum, timely and thoughtful preparation is key to preventing a study design chosen primarily for economic or efficiency reasons from becoming a flaming fiasco. Our BBH experience has shown that strong and nimble biostatistics leadership, integrated into a robust investigative team infrastructure, has the potential to overcome research study challenges and realize a research program’s overall goals by orchestrating necessary modifications while simultaneously preserving data integrity to achieve all study aims and advance the science of the field.

## Data Availability

The datasets generated and/or analyzed during the current study are not publicly available due their use for quality assurance during the ongoing data collection of BBH but are available from the corresponding author on reasonable request.

## References

[1] Mehta CC, Hagen KS, Rubtsova AA, Lahiri CD, Michopoulos V, Moran CA, et al. Bone, Brain, Heart Study Protocol: A Resilient Nested, Tripartite Prospective Cohort Study of the Role of Estrogen Depletion on HIV Pathology. Under revision PLOS ONE. in print. 10.1371/journal.pone.0272608 PMC9348736 35921353

[2] Adimora AA, Ramirez C, Benning L, Greenblatt RM, Kempf MC, Tien PC (2018). Cohort Profile: The Women's Interagency HIV Study (WIHS). Int J Epidemiol.

[3] Barkan SE, Melnick SL, Preston-Martin S, Weber K, Kalish LA, Miotti P (1998). The Women's Interagency HIV Study WIHS Collaborative Study Group. Epidemiol.

[4] Bacon MC, von Wyl V, Alden C, Sharp G, Robison E, Hessol N (2005). The Women's Interagency HIV Study: an observational cohort brings clinical sciences to the bench. Clin Diagn Lab Immunol.

[5] White S. Basic and Clinical Biostatistics. 5th ed. New York: McGraw-Hill; 2019.

[6] Rothman KJ (2002). Epidemiology: An Introduction.

[7] Hudson JI, Pope HG, Glynn RJ (2005). The cross-sectional cohort study: an underutilized design. Epidemiol.

[8] Gail MH, Altman DG, Cadarette SM, Collins G, Evans SJ, Sekula P (2019). Design choices for observational studies of the effect of exposure on disease incidence. BMJ Open.

[9] Gerstman BB (1998). Epidemiology Kept Simple: An Introduction to Classic and Modern Epidemiology.

[10] D'Souza G, Bhondoekhan F, Benning L, Margolick JB, Adedimeji AA, Adimora AA (2021). Characteristics of the MACS/WIHS Combined Cohort Study: Opportunities for Research on Aging With HIV in the Longest US Observational Study of HIV. Am J Epidemiol.

[11] Lesko CR, Buchanan AL, Westreich D, Edwards JK, Hudgens MG, Cole SR (2017). Generalizing Study Results: A Potential Outcomes Perspective. Epidemiology.

[12] Lesko CR, Buchanan AL, Westreich D, Edwards JK, Hudgens MG, Cole SR (2018). Generalizing Study Results: A Potential Outcomes Perspective: Erratum. Epidemiol.

